# Child sexual abuse in Iran: a systematic review of the prevalence, risk factors, consequences, interventions and laws

**DOI:** 10.5249/jivr.v14i3.1754

**Published:** 2022-07

**Authors:** Morteza Danaeifar, Maliheh Arshi, Amir Moghanibashi-Mansourieh

**Affiliations:** ^*a*^ Department of Social Work, University of Social Welfare and Rehabilitation Sciences, Tehran, Iran.

**Keywords:** Child sexual abuse, Child abuse, Child protection, Iran

## Abstract

**Background:**

Child sexual abuse is a widespread global problem and a violation of human rights. Although many studies have been conducted in this field in the world, the information and knowledge of child sexual abuse in Iran is still limited. This study aims to review the current knowledge in the area of child sexual abuse in Iran, and the related laws.

**Methods:**

The research systematic review covers the scientific literature and gray literature in Persian and English in Iranian and international databases from the beginning to June 2021 as well as Ira-nian laws on child protection.

**Results:**

Our study shows that in Iran knowledge on child sexual abuse is limited. The prevalence of child sexual abuse is estimated to be 1.5 to 32.5%; the risk factors for child sexual abuse encompass substance abuse, low literacy and education, parents living separately and divorce, poverty and poor socioeconomic status, and living in large families. The consequences of child sexual abuse are anxiety, depression, and social problems. Effective local interventions focused on parents and abused children have been conducted to raise awareness and prevent psychosocial harms as well as reduce aggression and physical and mental problems of chil-dren. Existing laws do not specifically address child sexual abuse.

**Conclusions:**

The findings showed that knowledge of child sexual abuse in Iran is limited, scattered and in-consistent and there is no suitable definition and tool for measuring child abuse in Iranian stud-ies. National and effective interventions for the prevention of child sexual abuse have not been performed and the consequences of child sexual abuse have also not been well studied. Thus further studies are required to estimate the prevalence of child sexual abuse at the national level and to assess the factors related to child abuse, its consequences, prevention methods and development of existing laws and policies with a special focus on child sexual abuse.

## Introduction

Child sexual abuse as a global problem and a violation of human rights^1^ includes a wide range of activities such as sexual caress, child exposure to adult sexual activity, child participation in prostitution or pornography, and sexual intercourse.^2^ Children are sexually abused by other children and adults, depending on their age or stage of growth, who are in a position of responsibility, trust or power over the victim.^3^ Child sexual abuse has adverse health, psychological and economic consequences for the victim and individuals in the society.^4^ Sexually abused children have been reported to have a wide range of aggressive behaviors or to suffer from several psychiatric disorders, including depression, anxiety, low self-esteem, unrestrained and suicidal thoughts, and are also at greater risk for substance abuse.^5-8^ These disorders depend on several factors, including the age of the child, the age of onset of sexual harassment, the time of the last harassment, and the relationship between the abuser and the abused.^9^

Collecting valid and evidence-based data on the prevalence of child sexual abuse is the first step to realize the nature and extent of the problem, to develop prevention and monitoring strategies and to assess the effectiveness of measures.^3^ Information on rates of sexual violence against children is not available, especially in developing countries, for a variety of reasons including sensitivity, shame, guilt, stigma, victim's lack of awareness of their own rights, fear of lack of proof, cultural issues, and non-disclosure of sexual harassment.^1,10,11^ Even the existing prevalence is different depending on the definition, the study design and sampling method and other methodological factors such as the type of questionnaire and the process of interviewing and obtaining information.^2,12^ However, a meta-analysis study estimated the global prevalence of child sexual abuse 127 cases per 1000 people. It also estimates the lifetime prevalence of child sexual abuse at 21.2% for women and 10.7% for men.^13^ According to a study, Africa and Asia are among the regions with the highest rates of child sexual abuse (CSA) in the world. They also share the highest proportion of children under 18 with Asia alone accounting for 24% of children under 14 years of age and about 41% of the African population are under the age of 15. Despite the magnitude of the problem, few studies have investigated CSA in these regions hence limiting appropriate and timely response. Prevalence of CSA (broadly and narrowly defined) in Africa to be from 2.1% - 68.7% for females in Tanzania and Ethiopia and 4.1% - 60% for males in South Africa. The prevalence in Asia ranges from 3.3% - 42.7% for females in China and India respectively and 4.3% - 58% for males in Hong Kong and Sri Lanka. The rates for contact CSA among females in Asia are from 1.9% - 59.2% in China and India and 1.8% - 9.1% for males in China while for non-contact abuse ranges from 1.8% - 28.7% for females in China and India and 3.1% - 29.4% for males in China.^14^ Some studies have estimated the prevalence of child sexual abuse between 2 and 62 percent in women and 3 to 16 percent in men based on the definition used and the population studied.^15^ According to a 2014 UNICEF report, 120 million girls worldwide have been the victim of sexual harassment at some point in their lives.^12^ Overall, the findings of previous studies show that women have been reported to experience two to three times more child sexual abuse than men.^8^

A review of the available literature shows that the study of the prevalence of child abuse in Iran is limited, previous studies have not directly examined child sexual abuse due to existing sensitivities and cultural issues, and few conducted studies have addressed the prevalence of children physical, emotional and inattention abuse. For instance, research conducted in Tehran reported a prevalence of children physical, emotional and inattention abuse of 17.5, 36.4 and 49.46%, respectively.^16^ Another study in Zanjan indicated the prevalence of emotional, physical and neglect abuse is 78, 56 and 39%, respectively.^17^

Child sexual abuse as a social issue based on the prevalence and the large number of associated consequences, in the short and long term, requires effective interventions and preventive strategies. Due to the lack of evidence relevant to the prevalence study at the national level and realization of social and cultural context of child sexual abuse in Iran, the purpose of this research is to systematically review the current knowledge in the field of child sexual abuse in Iran, along with the related laws, to develop knowledge in this area and to provide improvement of current policies as gaps are identified.

## Methods 

In this systematic research, to understand child sexual abuse in Iran, scientific sources and gray literature published in Persian and English, as well as laws related to child sexual abuse, were reviewed according to PRISMA standard criteria.^18,19^ In addition, manual and automatic search in Iranian and international online scientific databases were conducted to find published scientific materials, including articles, books, as well as gray literature to find unpublished articles, dissertations and publications of governmental and non-governmental organizations. 

An open-ended set of criteria was considered to obtain available articles and material,^20^ for example, any study or material was reviewed related to the prevalence, consequences, risk factors, prevention, or intervention related to child sexual abuse (under 18 years) in Iran which was published in prestigious scientific journals. A checklist was prepared to assess the quality of the methodology and design standard of the studies to ensure the quality of the collected materials. Excluded articles included systematic reviews, Meta-analyzes, commentaries, letters to the editors, non-Iranian studies, and studies on sexual harassment over the age of 18. All government and laws related to child sexual abuse were collected from websites and the Iranian Parliament.

**Search Strategy**

The search was performed by combining all terms associated with child sexual abuse, including violence, abuse, harassment, misbehavior and their equivalents along with the word "Iran", with the help of medical subject headings (MeSH). English databases of Web of Science, Scopus, PubMed, ProQuest and Google Scholar and Iranian databases including Magiran, IranDoc, SID were searched from the beginning to June 2021 and in the period from 12 to 19 June 2021 in accordance with the keywords formed in the review of English texts.

Inclusion and exclusion criteria: Every study or article related to the prevalence, consequences, risk factors, prevention and intervention related to child sexual abuse in Iran, published in reliable scientific journals in Persian or English language, was examined. All government laws related to child sexual abuse were also collected from the Iranian Parliament website. The articles that were excluded from the study included systematic review articles, meta-analyses, commentaries, letters to the editor, non-Iranian studies, and the study of sexual abuse at the age older than 18 years.

**Search results**

**Figure 1:**
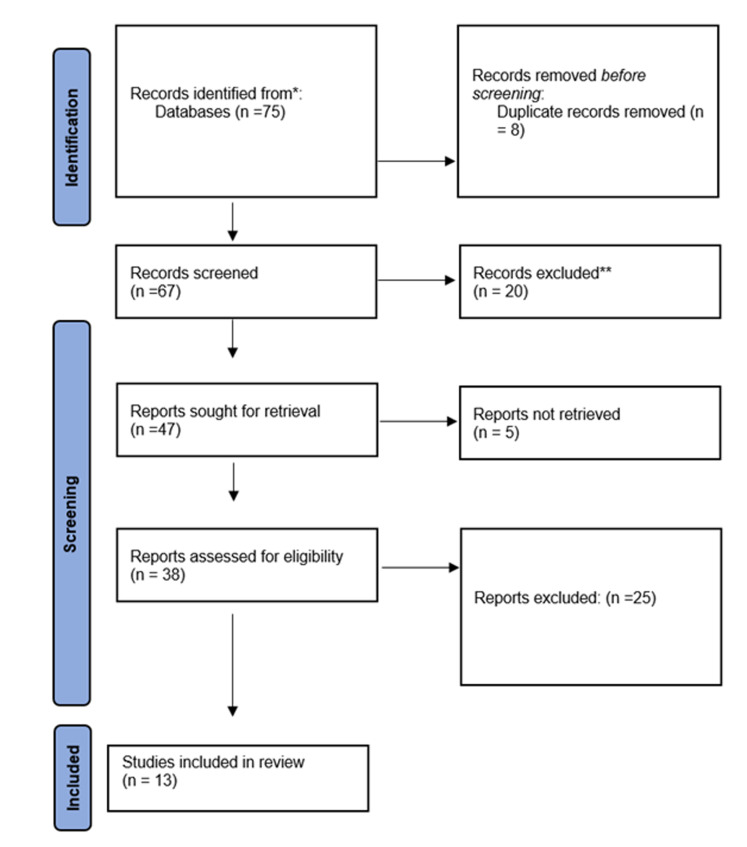
Report of searched and remaining articles based on PRISMA flow diagram.

The total number of articles obtained in the databases was 75, of which 17 were in Iranian databases, 2 in ProQuest, 6 in PubMed, 5 in Scopus, 2 in Web of Science and 43 in Google Scholar. Of these, 8 articles were duplicates and were deleted, and the number of results before quality evaluation was 67. After evaluating the quality based on the checklist, 54 articles were deleted due to non-relevance. A review of existing laws also revealed that only three legal documents directly or indirectly addressed child sexual abuse. Thus, at the end, 13 articles and 3 legal documents remained for review (Figure 1, PRISMA flow diagram).

**Selection of the research and data collection**

Titles and abstracts of all articles identified in Persian and English databases were screened independently by the first and second authors of the article, and each article that met the inclusion criteria was re-read the full text by two authors. The information of eligible articles was entered in the study data extraction form at this stage.

**Data Extraction**

We extracted the following data from the articles that met the inclusion criteria: 1- Article title 2- First author 3- Year of publication 4- Methodology 5- Sample size 6- Findings 7- Study language (Table 1).

**Table 1:** Characteristics of Iranian studies on child sexual abuse

Author, Year	Place	Sample Size	Methodology	Results	Language
Fakhari, 2012^21^	Tabriz	380	Cross-sectional	Prevalence of child sexual abuse in girls 2.3%	English
Namdari, 2004^22^	Khorram Abad	240	Cross-sectional	Prevalence of child sexual abuse in girls 32.5% (first, second and third grade of guidance school)There is a link between family financial status, education, addiction and parental illness with child sexual abuse	Persian
Nilchian, 2012^23^	Isfahan	301	Case-note review	The prevalence in both genders 4.1%	English
Derakhshanpour, 2017^24^	Bandar Abbas	68	Quasi-experimental	Prevalence of child sexual abuse 1.5%The effect of psychosocial intervention on the interaction between children and parents	English
Pirdehghan, 2015^25^	Yazd	700	Cross-sectional	The prevalence of child sexual abuse in both genders 28.8% in women 31.5 and in men 25.3% Addiction, education and parents' life together are among the factors affecting child sexual abuse	English
Nilchian, 2012^26^	Isfahan	25	Qualitative approach	Poverty, addiction and divorce were among the factors associated with child sexual abuse	English
Malahi, 2019^32^	Tehran	30	Quasi-experimental	Gestalt play therapy has been effective in reducing aggression and increasing social skills	Persian
Momayezi et al, 2011^34^	Tehran	144	Descriptive-correlational	Relationship between aggression and social skills with being abused	Persian
Jabber Qaderi et al 2010^33^	Tehran	14	Quasi-experimental	Effect of desensitization through eye movement and reprocessing and immunization against stress in improving mental problems of sexually abused children	Persian
Soltani Sarvbala 2013^27^	Tehran	400	Descriptive-correlational	Sexually abused children are more likely to live in large families, one-third live in single-parent families, have illiterate parents, are not in a good socioeconomic status, and have addicted and imprisoned parents	Persian
Azadeh Heidari 2011^31^	Tehran	307	Descriptive-correlational	Sexually abused children suffer from disorders of depression, anxiety, social problems, and thinking	Persian
Heidarzadeh 2018^29^	Tehran	200	Descriptive-analytics	There was no relationship between father's education, income status, father's addiction and parents' employment with child sexual abuse, but type of mother's addiction was related to child sexual abuse	Persian
Mousavi Khorshidi 2014^28^	Mazandaran	42	Descriptive-correlational	There is a relationship between parents' addiction and their socioeconomic status with child sexual abuse	Persian

## Results

Table 1 shows the characteristics and descriptive findings of the studies.

Most research involved a case study of a small geographical location (such as a provincial or county case study) or focused on a small target group. This indicates that existing research has not yet provided sufficient knowledge about the national image of child sexual abuse in Iran. Findings from the reviews of all 13 articles and 3 legal documents can be presented in five sections: prevalence rate, risk factors, consequences, interventions and laws on child sexual abuse in Iran.

**Prevalence of child sexual abuse**

We found five studies that estimated the prevalence of child sexual abuse in different parts of Iran. The prevalence reported in these studies varied widely, ranging from 1.5% to 32.5%. A study conducted in Tabriz^21^ reported the prevalence of child abuse in girls as 2.3% and another study on a sample of middle school girls in Khorram Abad showed that 32.5% of girls were sexually abused.^22^ A study conducted in Isfahan^23^ reported the prevalence of child sexual abuse in men and women is 4.1%. Conducted research in Bandar Abbas^24^ estimated it as 1.5%. Finally, research conducted in Yazd^25^ estimated it in both genders at 28.8, in women 31.5 and in men 25.3%. Most studies had focused on girls’ sexual abuse,^21,22,25,26^ although two studies also reported it in boys.^25,26^

**Risk factors of child sexual abuse**

A review of the reviewed research^22,25,26,27,28^ suggests that poverty and socioeconomic characteristics are associated with child sexual abuse in Iran. Only one study^29^ has not reported a relation between income status and child sexual abuse. Poverty is associated with poor child welfare, and child sexual abuse is well established in societies where there is poverty. Poverty corresponds to low education, lack of information and less access to prevention and intervention resources and can increase child sexual abuse.^30^

As research reviews have suggested mental disorders of family members were associated with child sexual abuse,^24^ a review of preliminary studies suggests that behavioral, emotional, mental illness problems including depression, anxiety, low tolerance for failure, and low self-esteem are the characteristics of families with a sexually abused child. Disorders that affect the mood, way of thinking, judgment or perception, seem to play an important role in the phenomenon of child sexual abuse, because it impairs the knowledge and judgment of the abuser and makes them prone to child abuse.

Parental substance abuse was one of the other variables found in the studies reviews to be related to child sexual abuse. ^22,25,26,27,28^ Only one study^29^ showed that father drug addiction is not associated with child sexual abuse but the same study concluded that there is a relationship between mother drug addiction and child sexual abuse. Drug addiction is one of the most important factors that affects the personality of parents and encourages child abuse. As mentioned, previous studies have shown that child sexual abuse is associated with substance use and is one of the most important predictors of child abuse. 

Another factor that was found to be related to child sexual abuse is the life of the parents apart and in some cases their divorce.^25,26,27^ Divorce or separation of parents is the result of family incompatibility. Separation of parents affects the responsibility of caring and supporting children and makes children vulnerable, as a result the risk of child sexual abuse increases.

Most studies^22,25,27,29^ showed that there is a relationship between low literacy and education of parents with the occurrence of child sexual abuse. Other studies have shown that there is a relationship between parental imprisonment, living in a large family, and being a single parent^27^ with child sexual abuse, but one study^29^ reported no relationship between parental occupation and child sexual abuse.

**Consequences of child sexual abuse**

Only one study in Iran addressed the consequences of child sexual abuse, and the results indicate that children who have been sexually abused suffer from anxiety, depression, social and cognitive problems.^31^

**Reviewed research on conducted interventions on child sexual abuse**

A review of research indicates that no interventions on child sexual abuse have been nationally conducted. However, locally reviewed studies have suggested to conduct psychosocial interventions,^24^ group play therapy,^32^ desensitization through eye movement, and stress immunization training^33^ to improve parental awareness on child abuse, reducing aggression^34^ and physical and mental problems of abused children, as well as increasing the social skills of these children. The results of these studies indicate that psychosocial interventions and education have a positive effect on reducing annoying thoughts and physical and mental problems of children and also increase mothers' awareness of ways to prevent sexual harassment.

**Relevant laws on child sexual abuse**

A review of existing laws revealed that only three legal documents directly or indirectly address child sexual abuse:

1- Civil Law (Article 1041 of the Amendment of 2002): According to this article, marriage of a girl before reaching the age of 13 and a boy before reaching the age of 15 is subject to permission, but it will be at the discretion of the competent court provided that it is prudent.

This law indicates that the children may legally marry when younger than the International standard of 18 years for marriage referred to the General recommendation No. 31 of the Committee on the Elimination of Discrimination against Women and general comment No. 18 of the Committee on the Rights of the Child on harmful practices. According to official statistics, about 17% of girls in Iran get married before the age of 18.^35^ The consequences of determining the age of marriage under the age of 18 can be particularly severe for girls, as they are vulnerable to forced marriage, sexual intercourse, childbearing, domestic violence, sexually transmitted infections, rape and abuse. In addition, the law does not grant children any right to consent to marriage and sexual activity. However, according to the general recommendations of the Committee on the Rights of Children, States Parties of the Convention are required to determine the minimum age of consent for a person to have sexual activity. Therefore, it can be said that child sexual abuse in the form of a legal marriage will not be subject to any legal protection.

**Islamic Penal Code (Chapter 10 (Articles 88 to 95) and Articles 221, 224, 228, 244, 251, 658, 660 and 661) approved in 2013**

One chapter and 8 articles of this law is indirectly corresponding to the issue of child sexual abuse and can be used to protect sexually abused children. Of the strengths of this law, the reduction of punishment for criminals who were immature at the time of the crime and considered dissatisfaction of the immature girl's consent to sexual intercourse can be mentioned. Also, if sexual intercourse with an immature spouse causes the vagina to rupture and the urine and blood to flow out, then the full dowry, the full monetary compensation of the wife, and the loss of defloration of virginity must be paid by the couple. In addition, the alimony of wife is the responsibility of husband until the death of one of the spouses, even if he has divorced her. Of course, according to this law, different definitions are mentioned for an immature individual, and sexual satisfaction is defined only for extramarital affairs, and for extramarital affairs, if it is proven to the judge that immature girl does not have heart consent, it is considered as dissatisfaction. However, this is entirely up to the judge's taste. In addition, there is no mention of sexual satisfaction for boys.

3- Law on the Protection of Children and Adolescents (Articles 1, 9 and 10) approved in 2020: It can be said that the only law that specifically addresses child abuse in Iran. Article one of this law contains most generalities and definitions, in this section, a complete and comprehensive definition of children and their age is not provided. Even child sexual abuse is not defined as a specific term. Article 3 mentions the factors that lead to the intervention and legal protection of children and adolescents, in which there is no direct reference to child sexual abuse.

The second chapter highlights the executive structure of this law and the need to establish an office for the protection of children in the judiciary and at the provincial level to conduct studies and evaluations in order to enforce the law. In this chapter, the judiciary is allowed to intervene immediately to prevent victimization, but unfortunately no direct reference has been made to the type of intervention required after child sexual abuse. In various articles, this law obliges various organizations, including State Welfare, Law Enforcement, Prisons Organization, the Ministry of Interior, the Ministry of Cooperatives, Labor and Social Welfare, the Ministry of Health and the Radio and Television Organization, to support children and adolescents in accordance with the mentioned articles.

The third chapter addresses crimes and punishments that deprive children and adolescents of education or cause their problems and disabilities. Article 10 of this law also refers to the punishments related to sexual harassment in nine paragraphs. Finally, fourth chapter deals with the limits and responsibilities of social workers and judicial officials and department of justice in interventions on the committed crimes against children and adolescents.

To approve some provisions of this law, it is necessary to mention that the Law on the Protection of Children and Adolescents in the support of at risk and affected children has undergone changes and innovations in the prevention of the occurrence of crime against children and in the criminal investigation process and has been effective in promoting child support. These innovations place the new law in a good position to influence children in terms of cultural beliefs, so that it is expected the view that child is independent of their family and guardians and has independent rights will be strengthened and expanded in the process of time.

One of the important points in the law is the legal protection of children and adolescents up to age of 18, in other words, every person from birth to reaching the age of 18 is subject to the provisions of this law. It is also possible to report a crime after seeing or being informed of child abuse by all natural or legal persons, and they are found guilty despite the capability to report and take action, provided that this action does not pose a similar or more severe danger to them or others. According to the provisions of this law, it is no longer necessary for the abused person or their family to file a complaint to prosecute the criminal, and according to the law, the prosecutor is obliged to prosecute the case and even if the private plaintiff agrees, the case is required to be investigated and if charged with the crime, the necessary punishment should be imposed. Other positive points of this law include criminalizing the spectrum of child sexual abuse from touch to rape and paying special attention to children with physical and mental disabilities.

## Discussion

A review of studies shows five key findings about child sexual abuse:

The first is the extent of child abuse in the reviewed studies, ranging from 1.5 to 32.5, which seems to be the only two studies^22,25^ with a prevalence rate of 32.5 and 28.8%, respectively, estimated similar to international estimates, and the others were lower than expected. The findings indicate that the prevalence estimated in the present study is higher than the estimated prevalence in Japan^4^ with a range of 1.3 to 8.3% for women and 0.5 to 1.3% for men and China^14^ with 1 for men and 9% for women.

However, the prevalence reported in the present study is similar to the estimated prevalence in India^36^ with a range of 4 to 41% in women and 10 to 55% in men. In a survey study with a sample size of 2309 people in France,^6^ the prevalence of child sexual abuse in girls was 13.1% and in boys 4.2%. Also, in a cross-sectional study in Saudi Arabia^2^ on 10,156 people, the prevalence of child sexual abuse during life was 20.8%, which is lower than the estimates made in our study. Some studies reviewed in the present study, in line with other studies, have estimated the prevalence of child abuse in girls more than boys^37,38^ and two studies, contrary to the results of other studies,^36,39^ the prevalence of child sexual abuse in boys They have estimated more than girls. 

Of the reasons for different prevalence of child sexual abuse in Iranian and foreign studies, it can be mentioned low and non-random sample size, different sampling strategies, type of study, data collection tools, inappropriate and varied definitions of child sexual abuse, and culture of non-disclosure of sexual harassment in the studied samples. Most Iranian studies have been conducted on students in marginal and high-risk areas, so the reported prevalence is likely to be higher than the national average. Even low estimates in some of the reviewed studies can be attributed to fear of disrespect, shame, guilt and stigma, fear of punishment and blame by the family, and the gap between parents and children, which have been highlighted in other articles.^36,40,41^ According to Islamic law, permission to engage in sexual activity in Iran is subject to legal marriage, and unlike most countries in the world, Iranian law has not considered any age for consent to engage in sexual activity, therefore different child sexual abuse statistics In Iran compared to other countries, is because the proof of consent is challenging. Finally, it can be said that since few studies have been done in this area and scattered in different geographical areas, it is not exactly clear in which areas and among which gender, child sexual abuse takes place more.

A review of few studies which have estimated the prevalence suggests that the government has no plans to provide a reliable national picture of the prevalence of child sexual abuse. This could be due to the taboo nature of this issue in Iran and the lack of proper response to the instances and public discussion of families about sexual abuse on their children and hidings of being sexually abused by children due to fear, so researchers are less willing to investigate difficult issues and sensitive culture in an Islamic society.

Other studies have emphasized the importance of taboo child sexual abuse and keeping child sexual abuse secret among children and their families.^1,42^ Many children do not report sexual harassment for a variety of reasons, including guilt and fear,^43^ and some families do not disclose it even know about their child's sexual abuse, for reasons such as fear of disgrace or inability to prove the guilt.^44^ As a result, due to improper reporting, accurate statistics on child abuse rates are not formed, and without reliable reports on the extent of the problem, appropriate preventive measures and interventions are planned nationally or locally. It seems that this problem exists in Islamic countries, especially in Iran.

Second, the findings of the present study showed that poor socioeconomic status and poverty in general are associated with child sexual abuse. In line with the present study, other studies have also shown poverty and poor socioeconomic status are related to child sexual abuse, commercial sex, and children trafficking.^36^ Findings of the current research showed that mental disorders of family members are related to child sexual abuse. Consistent with the present study, other studies have shown that mental disorders of family members, so lack of adequate family support, make children vulnerable.^36,45,46^ In line with the findings of other studies,^36,47^ parents drug addiction was another variable that was found in the research review to be related to child sexual abuse. Also, the present study, similar to other studies,^1,36^ indicated that parents' low education is associated with the experience of child sexual abuse. Another finding of this research which is consistent with other studies,^1,36^ is that divorce and separate parents are associated with child sexual abuse. Only one of the studies reviewed the perpetrators of sexual harassment, and its findings indicate that most sexual harassment was carried out in the family environment and by relatives who are older than the victim. This finding is consistent with other studies.^36^

Third, the review suggested that, unfortunately, only one study in Iran has been conducted on the health consequences of child sexual abuse, and its findings indicate that depression, anxiety, and social problems are more common in sexually abused children than in other children. This result is in line with the findings of other studies.^36,48-51^ A study in Saudi Arabia^2^ found that people who were sexually abused as children were more likely to develop depression, anxiety, and other mental illnesses.

Fourth, the results of research reviewed on special interventions for child sexual abuse, indicate that psychosocial interventions and education have a positive effect on reducing annoying thoughts and physical and mental problems of children and also increase mothers' awareness of ways to prevent sexual abuse. Research review indicates that the interventions conducted with a focus on abused children and carried out locally and no interventions were conducted to raise awareness and prevent child abuse at the local and national levels. As a result, it seems that there is a need for preventive policies and interventions based on culture in Iran in different groups of children, adolescents, parents, and teachers. Previous studies have shown that counseling, treatment, and educational interventions carried out on sexually abused children can generally reduce the symptoms of poor mental health^52^ and increase the knowledge and practices associated with sexual harassment.^53^ In general, it seems that the available interventions are at treatment, harm reduction and punishment of criminals and at the prevention levels of two and three, and this shows that interventions should be conducted at the basic level of prevention such as harassment prevention education and knowledge enhancement on sexual abuse.

Fifth, given that Iran is one of the countries committed to achieving sustainable development and eliminating violence against children, which was approved by the UN General Assembly in 2015, efforts have been made to review existing laws to protect children. Thus, the Law on the Protection of Children and Adolescents approved in 2002 has been revised and it has been approved by the Iranian parliament in 2020. But a review of past laws, and this law as the last law passed, revealed that the laws in Iran do not focus much on child sexual abuse. However, articles of the new law address the issue of child sexual abuse, its criminalization, the need for legal action and the organizations responsible for sexually abused children, and recognize that child sexual abuse is not limited to girls and boys are also subject to abuse and emphasize the protection of children and adolescents up to the age of 18. There are some deficiencies or shortages in the law including: 1- The main guardian is not known at the time of child sexual abuse, who first identifies the criminal factors before the problem occurs and secondly, conducts early interventions and takes preventive and educational measures against sexual harassment at the society. 2- It is the overlap of duties and actions that this law has entrusted to organizations and institutions. Firstly, the organization in charge of the protection of children and adolescents is not clear, and coordination between organizations is the responsibility of which person or institution. 3- Contrary to expectations, the enactment failed to provide comprehensive aspects of child protection, and there are many challenges ahead considering the possibility of evading its implementation by government institutions, under the pretext of lack of regulations, budget, and facilities. In addition, the penalties for violating this law by organizations have not been mentioned and the role of Ministry of Education as the main responsible organization for education has not been seen. 4. Finally, the existing problems in the criminal punishments mentioned in this law, because given that the exact definition of sexual abuse is not provided in this law, the limits of sexual harassment cannot be fully determined, since the punishments predicted in this law are derived from the existing laws in the principles of Islamic punishment as the main reference.

In the Islamic Penal Code, it is also necessary to punish a wrongdoer, first to prove a sexual offense, which in many cases is difficult and the committed may be acquitted without punishment, because according to Articles 114 and 224 of the Islamic Penal Code, rape exceeds the divine limits, and if the culprit repents and the judge achieves it, despite the conditions stipulated in religion and law, the sentence will be removed or reduced. It should be noted that the punishments provided in this law should be based on evidence and in favor of children; because it seems that the punishments provided in this law cannot prevent the recurrence of child sexual abuse and guarantee the protection of the child. It is necessary to consider the compensation of material, psychological and emotional damages of the victimized child in the formulation and execution of punishments, and the legislator should determine mechanisms to prevent the child from being re-victimized. It should be noted again that since the laws and punishments on child sexual abuse in Iran are subject to Islamic penal laws, as a result the legislator has not complied to a large extent with the criminal penalties provided for in the international conventions and laws.^54^

In general, although according to Article 9 of the Iranian Civil Code, "the provisions of the treaty concluded in accordance with the Constitution between the Government of Iran and other states are the rule of law", Iranian law on most cases of child sexual abuse, especially by incest and child marriage is contrary to international treaties and is largely subject to domestic law, jurisprudence and religious law.

It is also mentioned that due to the lack of specialized courts for child abuse and lack of sufficient awareness, judges use the minimum penalties for the perpetrators. One of the reasons mentioned for this issue is the influence of judges on the prevailing cultural and religious attitudes and contexts, for this reason advocates of children's rights believe that the judge should enforce the sentences in a way that protects children from any sexual abuse. ^28,54,55^

**Recommendations: **In order to achieve the United Nations Sustainable Development Goals^56^ and the production of evidence-based and reliable data, in the absence of national evidence in Iran, it is necessary to estimate the types of child sexual abuse, especially online child abuse and on the web, using valid tools and at the national level. To achieve the experiences of abused children and their families, conducting qualitative research can also be useful, and based on the data obtained, preventive interventions to be formulated specific to the cultural, social, ethnic and religious situation of the country. Since there is no national program in Iran to protect children and in particular the prevention and intervention on child sexual abuse, it is necessary to formulate and implement interventions and programs to assess their effectiveness. In this regard, it seems necessary to identify the main guardian of children protection and support, joint cooperation between organizations, law enforcement and people for preventive interventions, community leaders and managers of organizations in charge of children's rights, social workers, teachers and religious leaders, medical staff should receive the essential education, and through this, the necessary information on child rights, child sexual rights and healthy sexual behavior should be provided to families and children themselves, and ultimately, in case of sexual harassment, specialized medical services, psychosocial, legal, and support to victims and their families to be offered. 

Finally, it seems necessary to inform children about sexual harassment through the media and schools and at the national level, so that it can be prevented before the problem occurs and even after the problem occurs, there are clear, transparent and comprehensive rules helps the victim to feel trusted and supported, and the perpetrator feels threatened and knows that he or she will be unforgivably punished if he or she commits the crime.

**Limitations:**

In this study, every effort was made to access all sources and articles, but unfortunately, due to Iran's lack of access to the PsycINFO site, the information of this publisher could not be retrieved which may have affected the results. The next limitation was the lack of initial studies reviewing the prevalence, factors, and consequences associated with child sexual abuse, so meta-analysis was not possible in this research. The interventions were carried out at the local level, but since a coherent and national study has not been conducted in Iran, as a result, it was not possible to evaluate the scientific effectiveness of these interventions. Most of the initial studies reviewed did not specifically address child sexual abuse, and most also investigated physical, emotional, and psychological child abuse. Also it is not clear exactly what kind of child sexual abuse has been reviewed as a sample. One of the strengths of this review study is the extensive investigation of scientific sources and gray literature and published laws related to child sexual abuse in Iran in Persian and English, thus despite the numerous limitations, this systematic review can contribute significantly to future research on child sexual abuse.

## Conclusion

To our knowledge, this research is the first systematic review that seeks studies in scientific sources and gray literature in Persian and English, thus it offers comprehensive information on the current situation of child sexual abuse in Iran. The findings showed that knowledge of child sexual abuse in Iran is limited, scattered and inconsistent and due to the inability to carry out the meta-analysis necessary assurance and also an accurate interpretation of the current situation cannot be provided. It was found that there is no suitable definition and tool for measuring child abuse in Iranian studies. National and effective interventions for the prevention of child sexual abuse have not been performed. The consequences of child sexual abuse have also not been well studied.

**Acknowledgments**

This research is part of a PhD thesis developed in collaboration with the University of Social Welfare and Rehabilitation Sciences.
